# Case of Double Vagina and Uterus, Etc.

**Published:** 1875-10

**Authors:** A. E. Hoadley

**Affiliations:** Chicago


### CASE OF DOUBLE VAGINA AND UTERUS,

### WITH PREGNANCY OF THE RIGHT UTERUS AND DELIVERY THROUGH THE LEFT VAGINA.

By A. E. HOADLEY, M.D., Chicago.

Mrs. S——, a large and robust woman, of German nationality, nineteen years of age, and in first pregnancy, summoned me on the 14th day of August, 1874, to attend her in confinement. I was informed that the patient had been experiencing regular and active labor pains for twelve hours. An examination revealed the fact that she had a double vagina and a double uterus, with pregnancy of the right uterus; the uteri lying side by side, with the two necks closely united, the vaginal septum extending from between them and terminating in a thick round cord, or fold of mucous membrane, just inside the vulva, so that the external genitals presented a normal appearance. On separating the labia, the outer end of the septum could be seen extending from the symphysis pubis to the fourchette, giving to each vaginal orifice the same shape and size.

Considering the case one of interest to the profession on account of its being very rare, I invited Dr. J. D. Skeer to visit the patient and examine the case with me. By the aid of the sound, the left or empty uterus was explored, which was expanded over the side of the pregnant or right uterus, and its cavity was fully six inches in length. The os of each uterus was very rigid and so closely contracted that it would barely admit the tip of the index finger, notwithstanding the use of the ordinary means to induce dilatation of the rigid os—such as pressure with the fingers previously covered with the ext. belladonnæ, inhalations of chloroform, and the use of opiates. It was not until the sixth day that dilatation commenced, the pains continuing regular and quite severe all the time.

Dr. W. H. Byford was called in consultation with Dr. Skeer and myself, and pronounced it a perfect case of double uterus and vagina. As the patient's strength remained comparatively good, he advised non-interference.

The presentation of the child from the first was normal, but as soon as dilatation had commenced I thought to hasten labor by turning, and as the membranes had not yet been ruptured, this was accomplished by external manipulation without the least difficulty, and the membranes ruptured. The breech then, under the influence of vigorous uterine contractions, descended rapidly, forcibly dilating the os, and at the same time rupturing the upper end of the vaginal septum, which afforded ample room for the passage of the child. Labor now progressed rapidly, and before I was aware of it, there was a foot of the child protruding through the rent into the left vagina. I found it impossible to return it, without completely rupturing the vaginal septum; but, without delay or difficulty, I succeeded in bringing down the other foot through the same orifice, and the labor, which was 138 hours in duration, was very soon terminated, leaving about two inches of the outer end of the vaginal septum unruptured.

The patient made a very rapid and perfect recovery, and in two months from her confinement visited my office. I made an examination of her genital organs, and by passing a sound into each uterus at the same time, I could demonstrate to my entire satisfaction, that the deformity was symmetrical, both uteri being of equal size, and that the rupture in the septum was closed and perfectly healed.

